# Conservative surgery with microwave ablation for recurrent bone tumor in the extremities: a single-center study

**DOI:** 10.1186/s12885-022-10233-y

**Published:** 2022-11-02

**Authors:** Kai Zheng, Xiu-chun Yu, Ming Xu, Jing-ming Wang

**Affiliations:** Department of Orthopedics, The 960Th Hospital of the PLA Joint Logistice Support Force, No. 25 Shifan Road, Jinan, 250031 China

**Keywords:** Recurrent bone tumors, Microwave ablation, Surgical treatment

## Abstract

**Background:**

Surgical treatment for recurrent bone tumors in the extremities still presents a challenge. This study was designed to evaluate the clinical value of microwave ablation in the treatment of recurrent bone tumors.

**Methods:**

We present 15 patients who underwent microwave ablation for recurrent bone tumors during the last 7 years. The following parameters were analyzed for outcome evaluation: general condition, surgical complications, local disease control, overall survival, and functional score measured using the Musculoskeletal Tumor Society (MSTS) 93 scoring system.

**Results:**

Percutaneous microwave ablation in one patient with osteoid osteoma and another with bone metastasis resulted in postoperative pain relief. Thirteen patients received intraoperative microwave ablation before curettage or resection, including those with giant cell tumors of bone (6), chondroblastoma (2), osteosarcoma (2), undifferentiated sarcoma (1), and bone metastases (2). All patients achieved reasonable local tumor control in the mean follow-up of 29.9 months. The functional score was 24.1 for the 15 patients 6 months after the operation. Four patients had tumor metastasis and died, whereas 3 patients with tumors survived, and the remaining 8 patients without the disease survived.

**Conclusions:**

Microwave ablation represents an optional method for local control in treating recurrent bone tumors in the extremities.

## Background

Surgical treatment of recurrent bone tumors in the extremities continues to present a challenge. Giant cell tumors of bone and chondroblastoma are common benign bone tumors prone to relapse [1, 2]. The tumor site is close to the articular surface, and most patients are young, impeding the reoperation of the recurrent tumor. The current literature suggests that the ideal treatment for giant cell tumors of bone and chondroblastoma is removing the tumor while preserving as much of the joint as possible [1, 2]. ﻿The re-recurrence rate of recurrent giant cell tumors of bone has been reported to be 58.8% for patients who had underwent curettage or intralesional resection without an adjunct treatment [3]. Moreover, 88% of patients with osteosarcoma recurrence are complicated with lung metastasis or other metastases, and the 5-year survival rate of these patients is about 9.5% [4]. Even if osteosarcoma relapses and metastasizes, more than 60% of the patients are still willing to undergo limb-salvage surgery [4]. Amputation provides no additional survival benefits [4, 5]. The complex surgical boundary of recurrent osteosarcoma increases the difficulty of limb-salvage surgery. Minimal trauma, reduced costs, and reasonable local tumor control should be considered in treating recurrent osteosarcoma.

As a thermal ablation method, microwave ablation has been widely used in the treatment of bone and soft tissue tumors [6–10]. Percutaneous microwave ablation has been successfully used to treat osteoid osteoma and bone metastasis in the extremities [11–13]. The process of microwave ablation in the operation of primary malignant bone tumors, invasive bone tumors, and bone metastases has also been reported [14–17]. ﻿Microwave ablation has been used to provide adequate local tumor control for ﻿juxta-articular osteosarcoma [18]. The technique has also been applied to treat skip metastases of osteosarcoma in the extremities [19].

Few studies have thus far investigated the clinical value of microwave ablation in recurrent bone tumors. In this study, microwave ablation was performed to treat recurrent bone tumors in 15 patients. Basing on this retrospective clinical study, we intend to explore the clinical value of microwave ablation in the treatment of recurrent bone tumors.

## Patients and methods

Microwave ablation was performed on 15 patients with recurrent bone tumors in the extremities at the 960th Hospital of the PLA Joint Logistics Support Force from January 2014 to June 2020. Data were collected from patient records, including surgical protocols and tumor recurrence and metastasis. All patients in this series were followed-up regularly and underwent postoperative limb function evaluation. Regular follow-up was performed in the outpatient department. Follow-up records included patients’ feelings, proprioception, imaging reexamination, and limb function evaluation, which was determined using the Musculoskeletal Tumor Society (MSTS) scoring system.

The inclusion criteria were as follows: (i) patients with recurrent bone tumors in the extremities; (ii) definite postoperative pathological diagnosis; and (iii) microwave ablation used to treat recurrent bone tumors. The exclusion criteria were as follows: (i) patients who died of other diseases; (ii) incomplete clinical, radiographic, and pathology report; and (iii) no standardized follow-up data.

### Surgical treatments

For patients with a recurrent bone tumor, surgical methods included percutaneous microwave ablation and intraoperative microwave ablation before tumor curettage and resection. The pathological type and location of the tumor should be considered in the resection and repair of bone defect. After intraoperative tumor microwave ablation, tumor curettage and bone graft were typically performed on chondroblastoma; tumor curettage and cementation were typically conducted on giant cell tumors of bone; and tumor resection and cementation were generally performed on malignant tumors. Internal fixation was used to increase bone strength in some patients.

Survival status was evaluated according to both local and distant tumor control. Reexamination of all patients was requested monthly for half a year after surgery, every 3 months between 0.5–2 years, every 6 months between 2–5 years, and annually after 5 years. Local disease control and metastases were recorded. Local recurrence was suspected initially based on abnormal symptoms of pain and swelling, evidence of new mass upon physical examination, and imaging studies. A biopsy was further performed to confirm the suspicion.

Pulmonary metastasis, bone metastasis, retroperitoneal metastasis, and other metastases were recorded in this study. Pulmonary metastasis was confirmed by chest CT, and retroperitoneal and bone metastases were confirmed by magnetic resonance imaging. The MSTS 93 score was used for functional evaluation at the preoperation, postoperation, and follow‐up in our study. Each of the 6 variables, including pain, function, emotional acceptance, and supports walking ability and gait, was assessed on a five‐point scale, giving a maximum score of 30 points. A higher MSTS score signified better functional results. Clinical complications, such as functional limitation, infection, pain, and swelling were recorded. Oncological failure was not recorded as a complication.

## Results

Clinical data for 15 patients with a recurrent bone tumor are summarized (Table 1). The pathological types of recurrent tumors included 1 case of osteoid osteoma, 6 cases of giant cell tumors of bone, 2 cases of chondroblastoma, 2 cases of osteosarcoma, 1 case of undifferentiated sarcoma, and 3 cases of bone metastasis. The series included 7 men and 8 women, with a median age of 27.5 y (range: 12–69 y). The median follow-up period was 27 months (range: 11–92 months). Microwave ablation was conducted on all patients in the following locations: femora, 9 (60.0%); tibiae, 3 (20.0%); humeri, 2 (13.3%); and radius, 1 (6.7%).

**Table 1:** Presents data of 15 patients; it is a case series study

**No**	**Gender/age (years)**	**Recurrent location**	**Tumor**	**Tumor site**	**Microwave use**	**Follow-up (months)**	Function* (Pre-/6 m)	**Recurrence** **or metastasis**	**Survival**
1	M/13	Femoral diaphyseal	Osteoid osteoma	Local only	Percutaneous ablation	31	24/30	No	Alive without tumor
2	F/39	Distal radius	Giant cell tumor		Ablation before curettage	92	20/28	No	
3	F/34	Proximal humerus				34	21/26	No	
4	F/28	Distal tibia				24	19/26	No	
5	M/27	Distal femur				44	18/27	No	
6	F/30	Distal femur				30	21/27	No	
7	M/23	Proximal femur		Local and lung		32	18/27	Lung metastasis	Alive with tumor
8	F/15	Distal femur	Chondroblastoma	Local only	Ablation before curettage	33	22/26	No	Alive without tumor
9	M/15	Distal femur				25	23/25	No	
10	M/23	Middle femur	Osteosarcoma	Local and lung	Ablation before resection	15	18/22	Lung metastasis	Dead of disease
11	M/22	Middle femur				12	16/20	Lung metastasis	
12	F/68	Distal femur	Undifferentiated sarcoma			29	15/22	Lung metastasis	Alive with tumor
13	F/69	Proximal tibia	Liposarcoma metastasis	Local and Multiple metastasis	Ablation before curettage	12	14/17	Lymphatic metastasis	Dead of disease
14	M/12	Proximal tibia	Osteosarcoma metastasis		Ablation before curettage	11	13/20	Lung metastasis	
15	M/63	Proximal humerus	Renal carcinoma metastasis		Percutaneous ablation	25	13/19	Multiple metastasis	Alive with tumor

^a^According to *MSTS* (﻿Musculoskeletal Tumor Society) 93 limb functional score, *pre* Preoperation, *6 m*: 6 months after operation, *M* Male, *F* Female﻿

### Surgical treatments

**Fig. 1:**
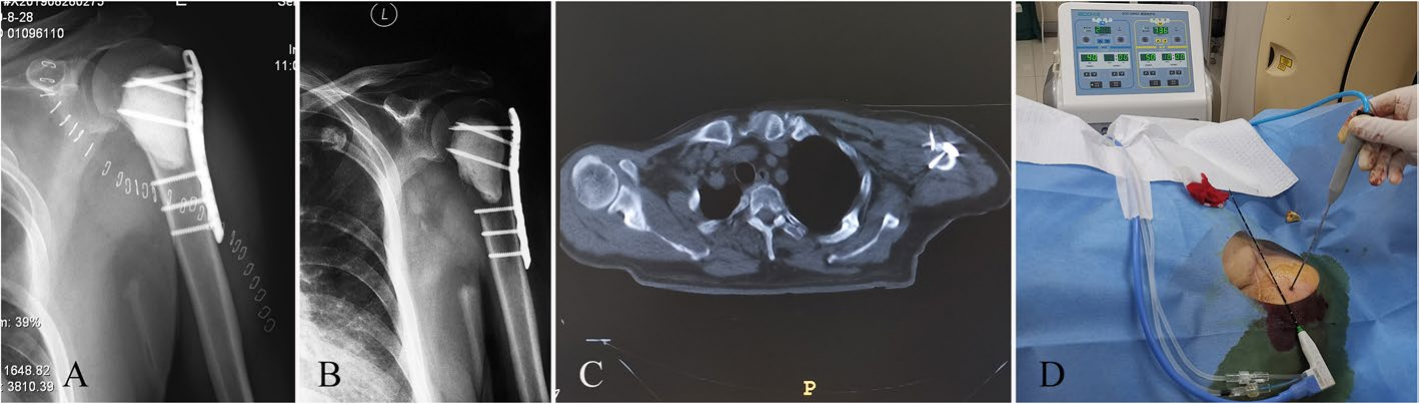
A 63-year-old male patient with ﻿with recurrent bone metastasis in the proximal humerus treated by percutaneous microwave ablation. **A** X-ray after the first surgery shows bone metastasis curettage, cementation and internal fixation. **B** X-ray at postoperative 8 months shows recurrent bone metastasis under the cement. **C** Percutaneous microwave ablation has been used to treat the recurrent bone metastasis. **D** After microwave ablation of the tumor, the temperature of the ablation core area can be measured to 73 ℃

﻿ Two patients—one with recurrent osteoid osteoma (Patient 1) and another with recurrent renal carcinoma metastasis (Patient 15)—underwent percutaneous microwave ablation for the tumor; meanwhile, the remaining 13 patients underwent adjuvant intraoperative microwave ablation. In the percutaneous microwave ablation, the tumor lesion was accurately located, and the puncture path was planned under CT guidance. A coaxial technique was used to guide the insertion of a 15G disposable microwave ablation antenna (2 mm in diameter, 150 mm in length, 2450 MHz generator, ECO -100A1 from Nanjing ECO Microwave System Co., Ltd.) to the tumor center. Patient 1, a 13-year-old boy with osteoid osteoma in the left femoral diaphysis, reexperienced pain symptoms. The boy underwent percutaneous radiofrequency ablation of the tumor 8 months before the aforementioned symptoms. Ablation with microwave output power 40 W was performed on recurrent osteoid osteoma for 2 min under local anesthesia and nerve block anesthesia. Patient 15, a 63-year-old male patient with renal carcinoma metastasis in the proximal humerus, reexperienced pain symptoms in his left upper arm. The patient underwent tumor curettage followed by cementation and internal fixation 6 months ago. Pazopanib treatment failed to achieve tumor control. Imaging revealed new osteolytic lesions with a diameter of about 2 cm in the internal fixation area. The existence of previous internal fixation provided good bone stability; thus, the recurrent humoral metastasis was treated by ablation with a microwave output power of 50 W for 4 min under local anesthesia (Fig. 1).

**Fig. 2:**
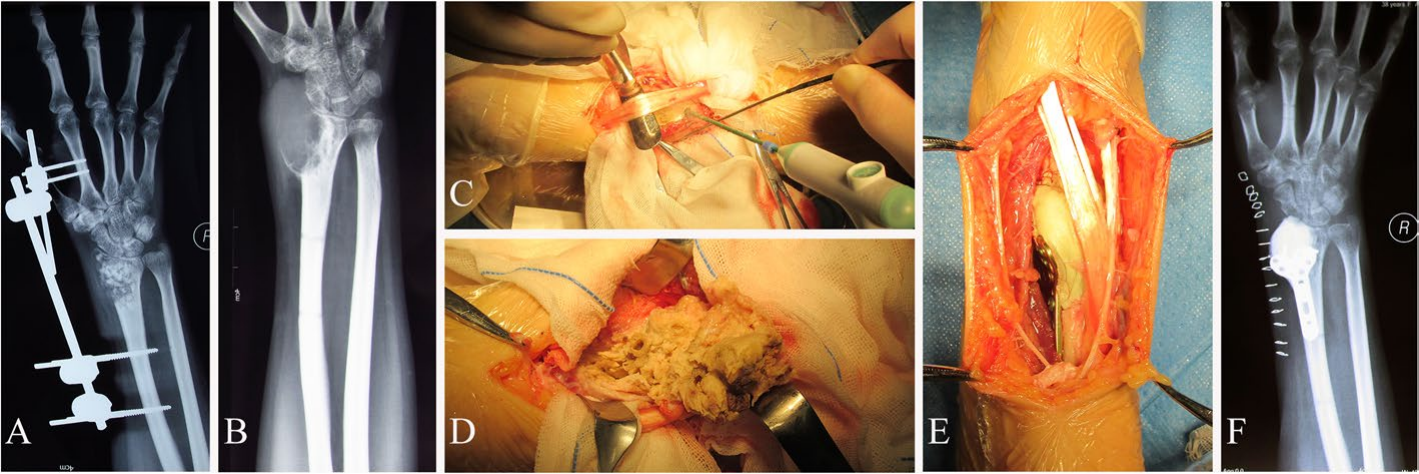
A 39-year-old female patient with ﻿with recurrent giant cell tumor of bone in the distal radius treated by tumor microwave ablation, curettage, cementation and internal fixation. **A** X-ray after the first surgery shows tumor curettage, bone-graft and external fixation. **B** X-ray at postoperative 4 months shows recurrent giant cell tumor of bone. **C** Recurrent bone tumor accepted microwave ablation before curettage. **D** After microwave ablation, the tumor tissue shows grayish white degeneration. **E** Cementation and internal fixation has been used to reconstruct bone defect after tumor curettage. **F** X-ray after re curettage shows tumor clearance and reconstruction

**Fig. 3:**
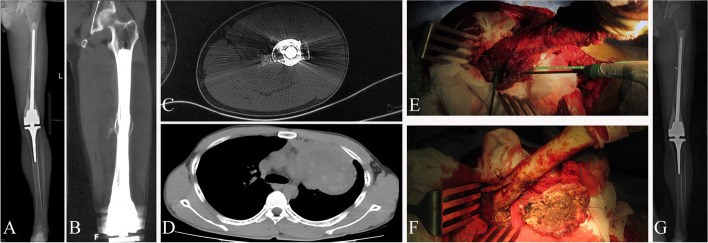
A 23-year-old male patient with ﻿with recurrent osteosarcoma in the middle femur treated by tumor microwave ablation and resection. **A**, **B**, **C** X-ray and CT show recurrent osteosarcoma in the middle femur at 3 years of after tumor bone inactivation combined with prosthesis reconstruction. **D** CT shows lung metastasis. **E** Recurrent osteosarcoma accepted microwave ablation before resection. **F** After microwave ablation, the degenerative tumor tissue accepted resection only. **G** Re operation without other treatments does not affect the stability of the prosthesis

Thirteen patients with local recurrent bone tumors underwent intraoperative microwave ablation before curettage or resection. The microwave equipment was the same as the aforementioned device. The microwave ablation power and ablation time were selected depending on the lesion location and size. When the microwave ablation area was close to the articular surface, intra-articular ice saline perfusion was employed. Six patients (Patients 2 to 7) with giant cell tumors of bone and 2 patients (Patients 8 and 9) with chondroblastoma underwent tumor curettage and bone grafting or cementation in the past 2 years. After recurrent tumor microwave ablation and curettage, 6 patients with giant cell tumors of bone received cementation (Fig. 2), and 2 patients with chondroblastoma was treated with allograft. ﻿The bone graft and cement in the primary treatment was totally removed before further curettage. Three patients (Patients 10, 11, and 12) with recurrent primary malignant bone tumors received intraoperative microwave ablation before resection. Patient 10 developed osteosarcoma recurrence and lung metastasis 3 y after tumor bone inactivation combined with prosthesis reconstruction (Fig. 3). Patient 11, who underwent osteosarcoma resection and mega-prosthesis reconstruction, had local recurrence and lung metastasis 9 months after surgery. Patient 12, who received undifferentiated sarcoma resection, experienced local recurrence and lung metastasis 6 y after surgery. She underwent microwave ablation, curettage, and internal fixation of a femoral tumor. Two patients (Patients 13 and 14) with recurrent bone metastasis were treated with intraoperative microwave ablation before curettage. Patient 13 had a left thigh tumor recurrence, left tibial metastasis, and multiple-lymph-node metastasis 19 months after resection of left thigh liposarcoma. Patient 14 had right tibial tumor metastasis 11 months after inactivation and replantation of osteosarcoma in the distal right femur.

Regarding the 2 patients who received percutaneous microwave ablation, for Patient 1, pain disappeared and the tumor was cured; for Patient 15, pain was relieved, and the VAS score was reduced from 9 to 2. Six patients (Patients 2 to 7) with recurrence after curettage of giant cell tumors of the bone regained local tumor control and retained their joints. One patient (Patient 7) received continuous denosumab treatment for lung metastasis. Two patients (Patients 8 and 9) with recurrent chondroblastoma regained local tumor control and retained their joints without other interventions in the follow-up. Four patients (Patients 10, 11, 13, and 14) with malignant recurrent bone tumors and multiple metastases achieved good local tumor treatment within the survival time. Patient 12 with a malignant recurrent bone tumor achieved good local tumor control and stable lung lesion in the follow-up.

In this series, 1 patient (Patient 7) with a giant cell tumor of bone and 6 patients (Patients 10 to 15) with malignant bone tumors had lung metastases or multiple metastases. Patients 7, 12, and 15 survived despite having tumors. The other 4 patients died of tumor metastasis 11–15 months after the operation. The mean MSTS score for limb function 6 months after the operation in 15 patients was 24.1 (range: 17–30). Compared with the mean score of 18.3 points of limb function before operation, the postoperative limb function has been improved to varying degrees. The limb function of the patients with benign tumors (25–30) was better than those of patients with malignant tumors (17–22) because of the differences in previous operations. Thirteen patients who received microwave ablation during operation, the mean operation time was 135 min (range: 85–160), and the average bleeding volume was 260 ml (range: 180–750). Except one elderly patient with preoperative anemia who received blood transfusion, other patients did not receive blood transfusion. No perioperative complications were found in these 15 patients.

## Discussion

﻿Percutaneous microwave ablation can effectively relieve pain and improve the quality of life of patients with benign bone tumors or malignant bone tumors [9]. Percutaneous microwave ablation of osteoid osteoma in the extremities is effective and has been recommended by clinical guidelines [20]. The current study proves that percutaneous microwave ablation can still achieve satisfactory clinical efficacy for osteoid osteoma with failed percutaneous radiofrequency ablation. For osteolytic limb bone metastasis, percutaneous microwave ablation can exert an analgesic effect. However, bone strength reduction cannot be improved, and pathological fracture poses a risk. The guideline recommends internal fixation for the osteolytic metastasis after microwave ablation [20]. In the present study, the patient with recurrent bone metastasis did not need to consider the risk of pathological fracture because of internal fixation. Thus, percutaneous microwave ablation can achieve the therapeutic effect. Percutaneous microwave ablation is a good treatment for similar cases.

The clinical effect of intraoperative microwave ablation has been proved in several studies [16, 21]. In the current study, the aforementioned technique was used to treat recurrent giant cell tumors of bone and chondroblastoma. Giant cell tumors of bone and chondroblastoma are found in younger patients. For recurrent bone tumors near the joint, bone tumor and joint resection, followed by mega-prosthesis reconstruction may easily be conducted. However, these young patients have long survival time and more activities. The best treatment should be to remove the tumor while preserving the joint of the patient. ﻿We sense that preserving the native articular surface is better than joint-sacrificing reconstructions. Giant cell tumors of bone and chondroblastoma are benign bone tumors that are prone to relapse. In the treatment of recurrent giant cell tumors of bone, ﻿intralesional procedures without any adjuncts such as the burring or Polymethylmethacrylate result in an unacceptably high re-recurrence rate of about 58.8% [3]. ﻿Recurrent giant cell tumors of bone can be treated by further curettage with additional burring and cementation with an acceptable re-recurrence rate of 21.7% [3]. As a new method, the microwave ablation pretreatment proposed in the present study can release tumor recurrence. The clinical efficacy proved that intraoperative microwave ablation is an optional surgical method for patients with recurrent giant cell tumors of bone and chondroblastoma.

﻿ Compared with benign bone tumors, recurrent malignant bone tumors are more difficult to treat. Relapsed osteosarcoma consists of hardly manageable entities with poor prognosis [22]. Although many other treatments exist, reoperation is an important treatment for recurrent tumors. Obtaining local tumor control, reducing surgical trauma, and lowering medical expenses are issues to considered in reoperation. In this study, a relatively eclectic surgical treatment was chosen. ﻿Microwave ablation as a method for tumor boundary control has been reported [18]. Intraoperative microwave ablation has been applied in recurrent tumor inactivation before resection in this study. Compared with wide resection and amputation, it involves relatively less surgical trauma and the same local control. In addition, microwave ablation has other advantages, such as reducing bleeding of blood rich tumors and inducing immunity. Of course, microwave ablation also has some disadvantages, such as: operation learning curve, risk of scalding surrounding tissues, etc. The clinical guideline is helpful to further understand the advantages and disadvantages of microwave ablation and help clinicians correctly use microwave ablation [20]. Within the limited survival time in this series, the local tumor control is satisfactory. One elderly patient with recurrent undifferentiated sarcoma in distal femur accepted microwave ablation obtain good tumor control also during more than 2 years of follow-up.

The limb function was the focus of this study because all patients underwent at least two operations. The limb function of patients is related to this conservative operation and the previous operation. ﻿For patients with giant cell tumors of bone and chondroblastoma, sparing the articular end of the affected bone allows patients to retain their native joints and ligaments, affording improved proprioception and joint function after reconstruction. Patients with benign tumors can obtain satisfactory long-term limb function. The functional results in this series may be ﻿attributed to the preservation of a stable periarticular structure and optimal bone construct.

﻿Certain limitations of this study require consideration. First, this study has a small number of patients and no control group; therefore, we could not perform a comparative assessment of our clinical results with those of wide resection alone or other methods of treating tumors, such as argon–helium cryoablation. Second, although the risk of local recurrence of giant cell tumors of bone and chondroblastoma is expected to decrease at 2 y, risk still exists to a certain extent, which may be evident with a longer follow-up. A minimum of 5 years follow-up is needed to be more certain of its safety. Third, no consensus has been reached on the appropriate time and energy of microwaves, which balance the intensity of ablation needed to adequately destroy the tumor and retain the normal bone and the articular cartilage. Clinical guidelines recommend that tumor ablation should reach an ablation temperature of 60 °C–80 °C and last for 30 min [20]. The gross degeneration of tumor tissue is typically used as a reference assessing the effect of microwave ablation. Nonetheless, the intraoperative experience of the surgeon is an important basis for guiding microwave use.

﻿In this series, no patient had local tumor recurrence, which suggests that conservative surgery with percutaneous microwave ablation or intraoperative microwave ablation can be a viable and safe option for selected patients with recurrent bone tumors in the extremities. This study proves that this concept is feasible and thus proposes a reliable scheme.

## Conclusions

Conservative surgery with percutaneous microwave ablation or intraoperative microwave ablation may be a viable and safe option for selected patients with recurrent bone tumors in the extremities. While a small sample size, excellent results were achieved in the current study. To strengthen and verify the conclusions larger, high-quality clinic trials are required.

## Data Availability

The datasets used and/or analysed during the current study are available from the corresponding author on reasonable request.

## Abbreviations

CT: Computer tomography
MSTS: Musculoskeletal Tumor Society
